# Effects of ventricular conduction block patterns on mortality in hospitalized patients with dilated cardiomyopathy: a single-center cohort study

**DOI:** 10.1186/s12872-016-0313-4

**Published:** 2016-06-13

**Authors:** Xiaoping Li, Rong Luo, Wei Fang, Xiaolei Xu, Guodong Niu, Yixian Xu, Michael Fu, Wei Hua, Xiushan Wu

**Affiliations:** Department of Cardiology, Sichuan Academy of Medical Sciences and Sichuan Provincial People’s Hospital, Hospital of the University of Electronic Science and Technology of China, Chengdu, Sichuan 610072 China; Cardiac Arrhythmia Center, State Key Laboratory of Cardiovascular Disease, Cardiovascular Institute and Fuwai Hospital, National Center for Cardiovascular Diseases, Chinese Academy of Medical Sciences and Peking Union Medical College, Beijing, 100037 People’s Republic of China; Temperature and Inflammation Research Center, Key Laboratory of Colleges and Universities in Sichuan Province, Chengdu Medical College, Chengdu, 610500 People’s Republic of China; Division of Cardiovascular Diseases, Mayo Clinic College of Medicine, Rochester, MN 55905 USA; Department of Cardiology, Lanzhou University Second Hospital, Lanzhou, Gansu 730030 People’s Republic of China; Department of Medicine, Sahlgrenska University hospital/Östra hospital, Gothenburg, Sweden; The Center of Heart Development, Key Lab of MOE for Development Biology and Protein Chemistry, College of Life Science, Hunan Normal University, Changsha, 410081 People’s Republic of China

**Keywords:** Ventricular conduction block, Dilated cardiomyopathy, Pulmonary hypertension, Survival, Prognosis

## Abstract

**Background:**

Ventricular conduction blocks (VCBs) are associated with poor outcomes in patients with known cardiac diseases. However, the prognostic implications of VCB patterns in dilated cardiomyopathy (DCM) patients need to be evaluated. The purpose of this study was to determine all-cause mortality in patients with DCM and VCB.

**Methods:**

This cohort study included 1119 DCM patients with a median follow-up of 34.3 (19.5–60.8) months, patients were then divided into left bundle branch block (LBBB), right bundle branch block (RBBB), intraventricular conduction delays (IVCD) and narrow QRS groups. The all-cause mortality was assessed using Kaplan-Meier survival curves and Cox regression.

**Results:**

Of those 1119 patients, the all-cause mortality rates were highest in patients with IVCD (47.8, *n* = 32), intermediate in those with RBBB (32.9, *n* = 27) and LBBB (27.1 %, *n* = 60), and lowest in those with narrow QRS (19.9 %, *n* = 149). The all-cause mortality risk was significantly different between the VCB and narrow QRS group (log-rank *χ*2 = 51.564, *P* < 0.001). The presence of RBBB, IVCD, PASP ≥ 40 mmHg, left atrium diameter and NYHA functional class were independent predictors of all-cause mortality in DCM patients.

**Conclusions:**

Our findings indicate that RBBB and IVCD at admission,but not LBBB, were independent predictors of all-cause mortality in patients with DCM.

## Background

Dilated cardiomyopathy (DCM), a leading cause of heart failure and arrhythmia, is a disease of the heart muscle characterized by ventricular dilation and impaired systolic function. The prognosis in patients with DCM is poor. However, the clinical spectrum is wide, and it is difficult for physicians to predict which clinical course an individual patient may follow.

Patients with DCM present with an increase in the QRS duration in the presence of a ventricular conduction block (VCB) [1–3]. There is controversy regarding the type of bundle branch block (BBB) that is associated with poorer outcomes in patients with heart failure (HF) [4–8]. Most studies indicate that left BBB (LBBB) is an independent prognostic marker, whereas right BBB (RBBB) is a weaker marker or not associated with a worse prognosis. Conversely, two studies recently showed that RBBB but not LBBB is associated with an increased 1-year and 4-year mortality risk in hospitalized patients with HF [9, 10]. Patients with intraventricular conduction delays (IVCDs) can also present with DCM, often without specifying the particular type of BBB. These patients also have worse clinical outcomes [1–3].

The prognostic implications of VCB in the long-term mortality of patients with DCM merit examination due to the lack of data on this issue. Therefore, in the present study, we evaluated the association of VCB patterns and all-cause mortality and compared the prognostic values of RBBB, LBBB, and IVCD in hospitalized patients with DCM.

## Subjects and methods

### Patients and follow-up

**Fig. 1:**
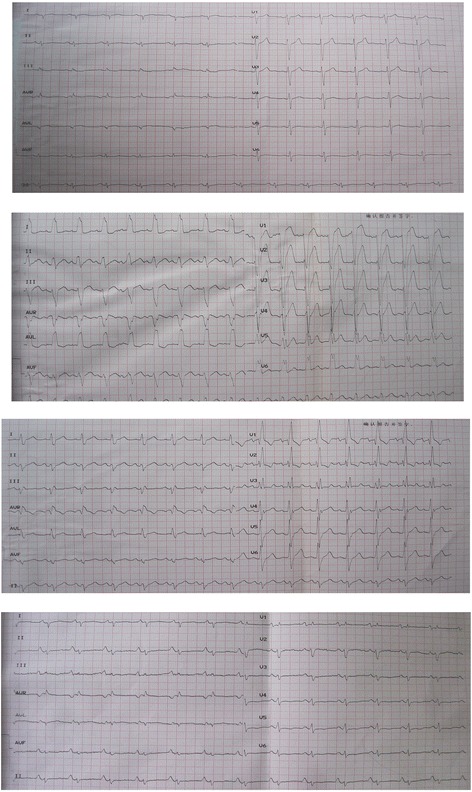
An example figure of narrow QRS, LBBB, RBBB, and IVCD

**Fig. 2:**
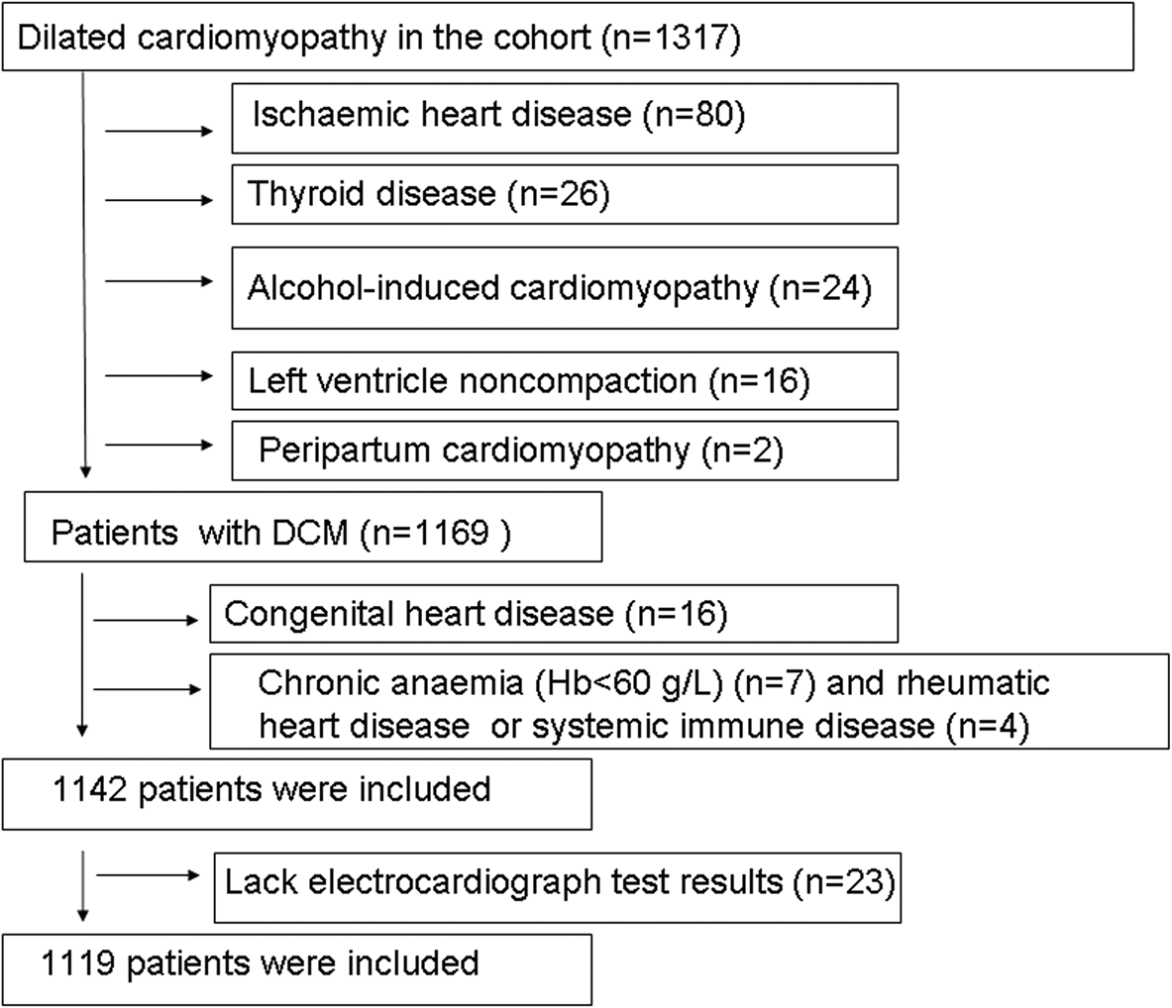
Derivation of the study cohort

This study was a retrospective, observational cohort study of patients with DCM observed from November 2003 to September 2011. VCB (LBBB, RBBB, IVCD) were identified from records of individual 12-lead ECGs in 1317 patients (Fig. 1). The patients were admitted due to their decompensation symptoms and the physical signs of heart failure, and DCM was defined as systolic dysfunction with LV dilation in the absence of an apparent secondary cause of cardiomyopathy [11]. We measured the following DCM exclusion criteria [12, 13]: systemic hypertension (>160/100 mmHg), coronary artery disease (>50 % in one or more major branches), chronic excess alcohol consumption (>40 g/day for females, > 80 g/day for males for more than five years after 6 months of abstinence), systemic diseases known to cause IDC, pericardial diseases, congenital heart disease, cor pulmonale, and rapid, sustained supraventricular tachycardia. Of the 1317 enrolled patients, 23 patients with missing electrocardiograph test results and 175 patients with various secondary cardiomyopathies were excluded from the study. The secondary cardiomyopathies included the following: 80 patients with ischemic heart disease by coronary angiography; 26 patients with overt hyper- and hypothyroidism thyroid disease; 24 patients with alcohol-induced cardiomyopathy; 16 patients with congenital heart disease; 16 patients with left ventricle noncompaction; 7 patients with chronic anemia (hemoglobin <60 g/L); 2 patients with peripartum cardiomyopathy; and 4 patients with rheumatic heart disease or systemic immune disease (Fig. 2). Thus, the final analysis included 1119 patients. The primary end point of the study was all-cause mortality, which was assessed for all patients through their medical records (patient’s hospital records, periodically examining the patient in the outpatient clinic) and medical follow-up calls with trained personnel. Data from patients who underwent cardiac transplantation were censored at the time of transplantation. The median follow-up period was 34.3 (19.5–60.8) months, and the study protocol was approved by the Ethics Commission of Fuwai Hospital.

### Echocardiography

The patients were imaged in the left lateral decubitus position using a commercially available system equipped with a 3.5 MHz transducer. Two-dimensional gray-scale, pulsed, continuous, color Doppler data were acquired from the parasternal and apical views. The left ventricular ejection fraction (LVEF) was calculated using the biplane Simpson’s technique.

Because pulmonary artery systolic pressure (PASP) is equal to the right ventricular systolic pressure in the absence of pulmonary stenosis, PASP was estimated using Doppler echocardiography by calculating the right ventricular to right atrial pressure gradient during systole (approximated by the modified Bernoulli equation as 4v^2^, where v is the velocity of the tricuspid regurgitation jet in m/s). Right atrial pressure, estimated based on the echocardiographic characteristics of the inferior vena cava and assigned a standardized value, was then added to the calculated gradient to give PASP. According to the new guideline, presence of PASP ≥ 40 mmHg was likely to be pulmonary hypertension (PH) [14].

### Statistical analyses

Continuous variables are expressed as the means ± SDs or medians and interquartile ranges. The categorical variables among groups were compared using chi-square (*χ*^2^) tests. Analysis of variance was used to compare continuous variables among multiple groups. Hazard ratios with 95 % confidence intervals were used to estimate the adjusted relative risk of the VCB groups. The Kaplan-Meier survival curves were compared using the log-rank test. Multivariate Cox proportional hazards regression models were used to adjust for any confounding variables among groups. First, the potential variables were evaluated by univariate analysis and were then selected based on their clinical and statistical significance. Second, a multivariate analysis was performed using Cox proportional hazards regression modelling adjusted for baseline variables. SPSS version 16.0 software (SPSS, Chicago, Illinois) was used for all statistical analyses. All of the tests were two-sided, and a p value < 0.05 was used to determine statistical significance.

## Results

### Characteristics of the study population

The cohort consisted of 1119 patients with DCM: 298 (26.6) women and 821 (73.4) men; 1076 (96.2) were from the Han population and 43 (3.8 %) were from other races: the mean age was 51.1 ± 14.7 years. Of those, 19.8 (*n* = 221) had LBBB, 7.3 (*n* = 82) had RBBB, 6.0 (*n* = 67) had IVCD, and 66.9 % (*n* = 749) had narrow QRS. Table 1 summarizes the baseline clinical characteristics of the cohort. Among the patients with VCBs (LBBB, RBBB and IVCD) and narrow QRS, the number of women with RBBB was lower, and there was a lower frequency of a history of hypertension but a greater frequency of PASP ≥ 40 mmHg in patients with RBBB. Patients with LBBB were older, were predominantly male, had more frequent essential hypertension and had longer QRS durations, QT intervals and larger LV diameters. The patients with IVCD had higher levels of circulating bilirubin, larger left atriums (LAs), larger right ventricle (RV) diameters, longer PR intervals, and less use of beta blockers, aspirin and spironolactone during admission.

**Table 1:** Patient characteristics categorized by ventricular conduction block patterns

	All patients (*n* = 1119)	LBBB (*n* = 221)	RBBB (*n* = 82)	IVCD (*n* = 67)	Narrow QRS (*n* = 749)	*P* value
Age (years)	51.1 ± 14.7	57.2 ± 12.0	53.1 ± 14.7	52.3 ± 14.5	48.9 ± 14.9	<**0.001** ^a^
Female gender, n (%)	298(26.6)	89(40.3)	16(19.5)	15(22.4)	178(23.8)	<**0.001**
History						
Disease duration (years)	2(0.5–6)	4(1–9)	4(1–8)	3(1.5–6)	2(0.35–5)	**0.001**
Essential hypertension, n (%)	294(26.3)	73(33.0)	15(18.3)	15(22.4)	191(25.5)	**0.034**
Diabetes mellitus, n (%)	160(14.3)	24(10.9)	15(18.3)	6(9.0)	115(15.4)	0.142
Atrial fibrillation, n (%)	257(23.0)	29(13.1)	20(24.4)	11(16.4)	197(26.3)	<**0.001**
Smoker, n (%)	517(46.2)	93(42.1)	33(40.2)	31(46.3)	360(48.1)	0.293
Drinker, n (%)	363(32.4)	59(26.7)	28(34.1)	22(32.8)	254(33.9)	0.243
NYHA class III and IV^c^, n (%)	817(73.0)	162(73.3)	60(73.2)	54(80.6)	541(72.2)	0.532
Admission vital signs						
SBP (mm Hg)	113.0 ± 17.7	114.6 ± 17.3	110.2 ± 18.8	111.7 ± 19.8	113.0 ± 17.4	0.242
DBP(mm Hg)	72.5 ± 12.6	71.8 ± 12.4	71.8 ± 11.1	69.3 ± 12.4	73.0 + 12.8	0.099
Heart rate, beat/min	80.9 ± 17.4	78.3 ± 15.9	79.6 ± 15.5	78.6 ± 14.4	82.0 ± 18.2	**0.023**
Laboratory values at admission^b^						
TB (mmol/L)	26.2 ± 19.6	24.9 ± 19.1	25.3 ± 16.0	34.8 ± 26.7	26.0 ± 19.3	**0.004**
DB (mmol/L)	3.7(2.5–6.5)	3.2(2.15–6.05)	3.5(2.7–7.4)	4.9(3.2–9.2)	3.7(2.5–6.485)	**0.003**
Glucose (mmol/L)	5.62 ± 1.85	5.86 ± 1.94	5.50 ± 1.52	5.34 ± 1.44	5.59 ± 1.89	0.150
Triglyceride (mmol/L)	1.57 ± 1.02	1.63 ± 0.99	1.58 ± 0.94	1.38 ± 0.70	1.56 ± 1.06	0.410
Total cholesterol (mmol/L)	4.61 ± 1.13	4.75 ± 1.12	4.52 ± 0.98	4.39 ± 1.23	4.59 ± 1.14	0.125
Creatinine (μmol/L)	92.8 ± 35.2	91.0 ± 26.4	94.9 ± 25.9	97.9 ± 40.7	92.6 ± 37.8	0.526
BUN (μmol/L)	7.95 ± 3.97	7.88 ± 2.67	8.04 ± 2.69	8.89 ± 4.68	7.88 ± 4.32	0.262
CK-MB (IU/L)	13.5 ± 7.76	13.1 ± 6.77	12.0 ± 6.70	13.7 ± 7.59	13.8 ± 8.14	0.229
NT- Pro- BNP (fmol/mL)	2010.3 ± 1567.5	1998.0 ± 1595.8	2170.5 ± 1679.8	2358.4 ± 1638.4	1962.4 ± 1538.0	0.315
Electrocardiogram data						
QRS duration (ms)	119.6 ± 30.9	156.0 ± 24.4	153.3 ± 24.9	137.1 ± 23.1	103.6 ± 18.2	<**0.001**
QT (ms)	405.7 ± 54.2	434.4 ± 50.6	429.1 ± 51.1	421.3 ± 38.7	393.2 ± 52.4	<**0.001**
P (ms)	107.5 ± 21.6	102.7 ± 22.6	107.5 ± 23.6	108.2 ± 25.1	109.3 ± 20.3	**0.005**
PR (ms)	182.8 ± 32.9	184.9 ± 33.1	192.3 ± 42.0	193.6 ± 34.7	179.7 ± 30.7	**0.001**
Echocardiography data						
LV (mm)	68.0 ± 9.3	71.4 ± 11.4	68.7 ± 8.3	70.9 ± 13.1	66.7 ± 8.0	<**0.001**
LVEF (%)	31.9 ± 8.4	31.0 ± 8.2	31.8 ± 7.3	30.5 ± 9.0	32.3 ± 8.5	0.142
RV (mm)	23.6 ± 5.4	22.0 ± 5.0	24.1 ± 5.6	24.5 ± 5.5	24.0 ± 5.4	<**0.001**
LA (mm)	43.9 ± 7.7	43.1 ± 7.9	45.8 ± 8.4	46.6 ± 8.9	43.7 ± 7.5	**0.002**
PASP (>40 mmHg), n (%)	203(18.1)	35(15.8)	24(29.3)	15(22.4)	129(17.2)	**0.031**
Medicine during admission						
Diuretics, n (%)	1059(94.6)	205(92.8)	76(92.7)	63(94.0)	715(95.5)	0.362
ACEI/ARB, n (%)	951(85.0)	182(82.4)	70(85.4)	54(80.6)	645(86.1)	0.396
Beta-blockers, n (%)	1017(90.9)	195(88.2)	69(84.1)	56(83.6)	697(93.1)	**0.002**
Digoxin, n (%)	903(80.7)	168(76.0)	66(80.5)	59(88.1)	610(81.4)	0.127
Aspirin/anticoagulants n (%)	721(64.4)	131(59.3)	52(63.4)	34(50.7)	504(67.3)	**0.013**
Spironolactone, n (%)	1019(91.1)	191(86.4)	73(89.0)	57(85.1)	698(93.2)	**0.004**

^a^Data are expressed as the means ± SDs or medians (interquartile ranges) or as percentages, *P* values from an ANOVA or chi-square test for all four groups. Bold data indicated *P* <0.05

^b^Thirty-four patients lacked echocardiography data; 47 patients lacked data on PASP; 341 patients lacked NT-pro-BNP levels; 52 patients lacked fasting blood glucose levels; 46 patients lacked creatinine and BUN levels; 51 patients lacked CK-MB levels; and 89 patients lacked triglyceride and total cholesterol levels

^c^Abbreviations: *NYHA* New York Heart Association, *SBP* systolic blood pressure, *DBP* diastolic blood pressure, *TB* total bilirubin, *DB* direct bilirubin, *BUN* blood urea nitrogen, *CK-MB* heart-type creatine kinase isoenzyme, *PASP* pulmonary artery systolic pressure, *NT-pro-BNP* N-terminal fragment pro-brain natriuretic peptide, *LV* left ventricle, *LA* left atrium, *LVEF* left ventricular ejection fraction, *ACEI* angiotension-converting enzyme inhibitor, *ARB* angiotension receptor blocker

### Relation between VCB patterns and all-cause mortality

**Fig. 3:**
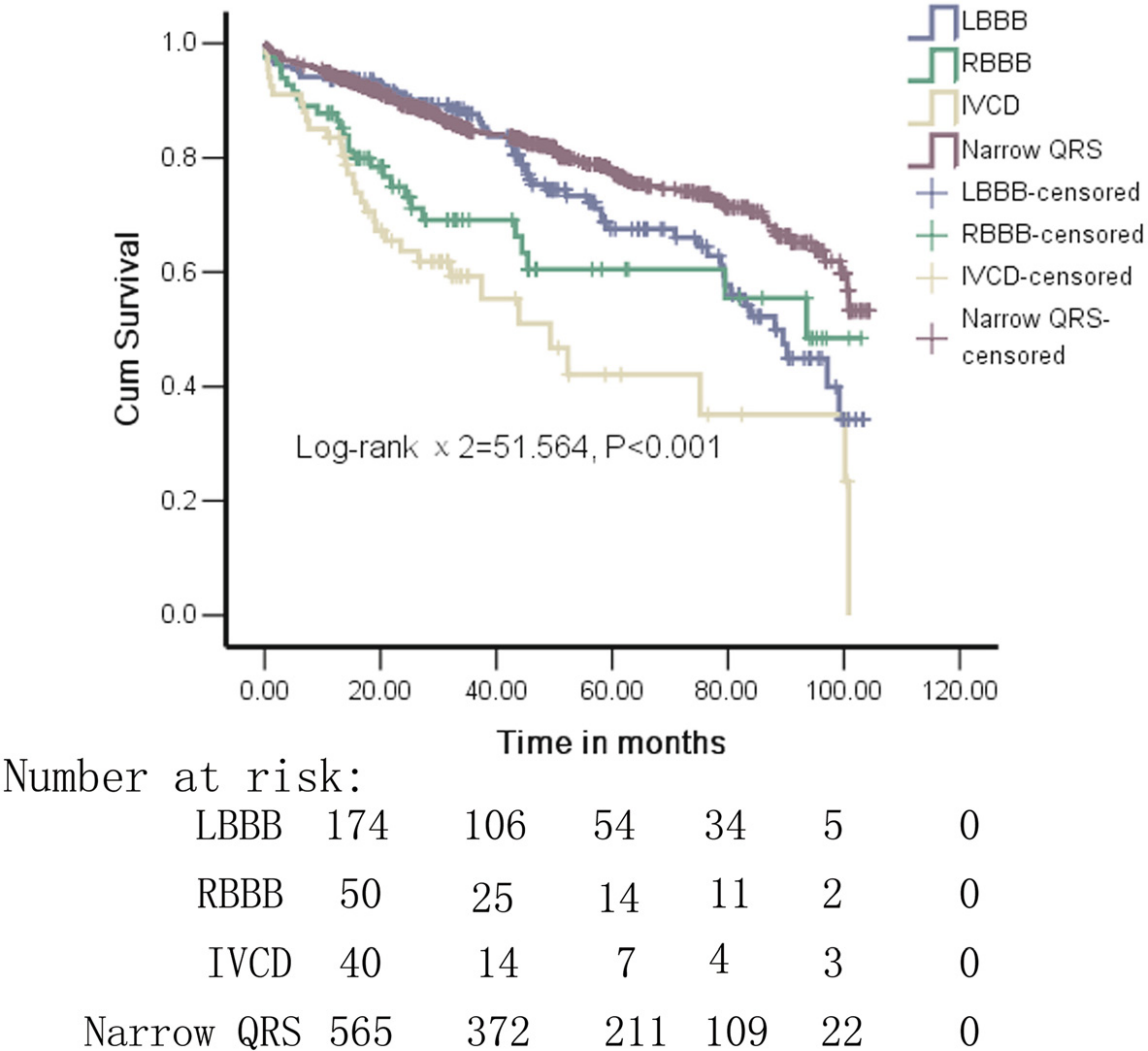
Kaplan-Meier survival curves for patients with DCM: patients with LBBB, RBBB, IVCD and narrow QRS (log-rank *χ* ^2^ = 51.564, *P* < 0.001). Abbreviations: DCM, dilated cardiomyopathy; VCB, Ventricular conduction block; LBBB, left bundle branch block; RBBB, right bundle branch block; IVCD, intraventricular conduction delay

Among the 1119 patients studied, 268 died and 3 underwent heart transplantation during a median follow-up of 34.3 (19.5–60.8) months. The all-cause mortality rates were highest in patients with IVCD (47.8 %, *n* = 32); intermediate in patients with RBBB (32.9, *n* = 27) and LBBB (27.1 %, *n* = 60); and lowest in patients with narrow QRS (19.9 %, *n* = 149). Over the median of 34.3 month follow-up, there was a significant difference in all-cause mortality risk between the VCB and narrow QRS groups (log-rank *χ*^2^ = 51.564, *P* < 0.001) (Fig. 3).

### Cox proportional hazard models

Table 2 summarizes the results of the Cox models in which each of the parameters were entered separately as the mortality explanatory variable. The univariate analysis indicated that age, history of essential hypertension and atrial fibrillation (AF), NYHA functional classes, disease duration, systolic blood pressure, diastolic blood pressure, LV, LA diameters, LVEF, PASP ≥ 40 mmHg, and the presence of LBBB, RBBB and IVCD were predictors of all-cause mortality in DCM patients. After adjustments for age, gender, history of essential hypertension and AF, smoking and drinking status, disease duration, blood pressure, heart rate, LV diameter and LVEF value, using either forward or backward selection, and the presence of RBBB, IVCD, PASP ≥ 40 mmHg, the NYHA functional class and LA diameter were the only variables that remained in the model and emerged as important predictors. However, unlike RBBB and IVCD, LBBB was not a predictor of death using the multivariate analysis.

**Table 2:** Cox-regression of all-cause mortality in patients with DCM

Variable	Univariate analysis			Multivariate analysis		
	HR	95 % CI	*P*-value	HR	95 % CI	*P*-value
Age	1.012	1.003–1.021	**0.007**	1.007	0.997–1.018	0.175
Gender	1.154	0.883–1.507	0.294	1.255	0.894–1.761	0.189
Essential hypertension	0.706	0.524–0.951	**0.022**	0.810	0.579–1.134	0.220
Atrial fibrillation	1.317	1.004–1.728	**0.047**	1.247	0.909–1.710	0.172
NYHA functional classes	1.619	1.376–1.905	<**0.001**	1.248	1.038–1.499	**0.018**
Disease duration	1.027	1.011–1.044	**0.001**	1.015	0.996–1.033	0.121
Smoker	0.973	0.850–1.114	0.691	0.972	0.819–1.154	0.746
Drinker	0.893	0.766–1.040	0.146	0.913	0.756–1.103	0.346
Heart rate	1.002	0.995–1.009	0.604	1.004	0.996–1.013	0.311
Systolic blood pressure	0.982	0.975–0.989	<**0.001**	0.992	0.981–1.003	0.148
Diastolic blood pressure	0.979	0.969–0.989	<**0.001**	0.989	0.974–1.004	0.136
Left ventricle	1.039	1.027–1.053	<**0.001**	1.016	0.998–1.016	0.078
Left atrium	1.055	1.040–1.071	<**0.001**	1.041	1.022–1.060	<**0.001**
LVEF	0.965	0.951–0.980	<**0.001**	0.984	0.966–1.003	0.093
LBBB	1.408	1.043–1.900	**0.025**	1.197	0.839–1.706	0.321
RBBB	2.091	1.387–3.154	<**0.001**	2.553	1.665–3.913	<**0.001**
IVCD	3.488	2.376–5.122	<**0.001**	3.726	2.417–5.745	<**0.001**
PASP ≥ 40 mmHg	1.992	1.529–2.596	<**0.001**	1.403	1.040–1.893	**0.027**

Note: The variables analyzed in the multivariate Cox mode included age, gender, the history of essential hypertension and atrial fibrillation, drinking and smoking status, disease duration, NYHA functional classes, systolic blood pressure, diastolic blood pressure, heart rate, left ventricle, right ventricle, left atrium diameter, LVEF, LBBB, RBBB, IVCD and PASP ≥ 40 mmHg. Bold data indicated *P* <0.05

## Discussion

In this study, we investigated the associations among different patterns of VCB and all-cause mortality in patients with DCM. Our major new finding suggests that RBBB and IVCD upon admission, but not LBBB, were strong predictors of all-cause mortality in patients with DCM.

Several studies investigating the predictive value of QRS morphology in patients with HF yielded conflicting results regarding mortality risk associated with the BBB pattern [4–7]. Baldasseroni et al. [5, 6] reported that complete LBBB, but not RBBB, was associated with a higher adjusted 1-year mortality rate in 5,517 outpatients with HF. McCullough et al. [4] found higher 2-year mortality rates for RBBB and LBBB compared with patients with normal QRS, but a multivariate analysis demonstrated that RBBB was not as powerful a predictor of mortality as LBBB. Most recently, Mueller et al. [7] analyzed the impact of the BBB pattern on long-term mortality and found that the mortality was significantly higher in HF patients with RBBB. Two studies recently showed that RBBB, but not LBBB, is associated with increased mortality risk in HF patients [9, 10]. None of these studies, however, reported the relationship between RBBB and mortality risk in the patients with DCM. In the present study, we found that RBBB and IVCD patients with DCM had a higher all-cause mortality than patients with LBBB, and patients with any pattern of VCB had higher all-cause mortality rates than patients with a narrow QRS. A multivariate analysis demonstrated that RBBB and IVCD, but not LBBB, were the predictors of all-cause mortality in patients with DCM.

Approximately 30% of patients with heart failure or cardiomyopathy have VCBs, such as left or right bundle-branch blocks [5, 9]. Some studies have shown that in patients with HF, the prevalence of LBBB is higher than in patients with RBBB [4, 5, 7]. LBBB is associated with more severe HF characterized by an advanced NYHA functional class and decreased LVEF, whereas RBBB is more prevalent in men and is not associated with advanced HF symptoms or ventricular dysfunction [5, 6]. In our study, the prevalence of VCB was 33.1 %; more patients had LBBB than RBBB or IVCD. Patients with LBBB had longer QRS durations, larger LV diameters and lower LVEF values than those with RBBB. However, patients with RBBB had more frequent of PASP ≥ 40 mmHg, along with larger RV diameters, than those with LBBB.

One of the reasons for a worse prognosis in patients with RBBB may be that they have an elevated pulmonary pressure compared with those with LBBB or a narrow QRS. Acquired RBBB is often associated with PH and right-sided cardiac failure, and PH complicated by heart failure is generally considered to be an indicator of a poor prognosis [11, 15]. In addition, right ventricular dysfunction has an additive predictive value in patients with left ventricular systolic dysfunction [16]. Furthermore, RBBB may be a marker not only of right ventricular dysfunction but also of severe intraventricular desynchronization of both ventricles. Recently, Fantoni et al. [17] reported that patients with RBBB had larger right ventricle electrical conduction delays compared with patients with LBBB using electromagnetic, catheter-based, 3-dimensional mapping. In the present study, compared with LBBB, patients with RBBB had more frequent of PASP ≥ 40 mmHg, larger RV diameters and higher all-cause mortality rates during follow-up.

Very limited data exists on patients with IVCD. Patients with myocardial infarction with IVCD had significantly greater interventricular asynchronies and higher BNP levels than post-myocardial infarction patients without IVCD [18]. In the Multicenter Unsustained Tachycardia Trial (MUSTT), patients with LBBB or IVCD had lower ejection fractions and a higher prevalence of congestive heart failure than those without these abnormalities. The presence of IVCD was associated with a 1.5-fold increased risk of cardiac arrest and total mortality in the patients treated with cardiac resynchronization therapy (CRT) [8]. In another study on heart failure with CRT, the all-cause mortality was also higher in patients with IVCD than LBBB or RBBB; the worst prognosis was seen in patients with IVCD [19]. The reason for the higher mortality rates in patients with IVCD is unclear, and further research is needed to confirm the role of IVCD in DCM.

The present study has several limitations. Like all hospital-based cohorts, this is a selected population of patients who have been referred for treatment. As with many studies of chronic diseases, the time of disease onset is not precisely known, and there may be variations in the length of the preclinical phase that influences the relationship between IVCD, PH and death. Because the N-terminal pro-brain natriuretic peptide (NT-pro-BNP) test was not commonly used until the later years of this study and was missing in 341 patients, we excluded NT-pro-BNP from the multivariate Cox analysis to avoid potential confounding variables in the statistical analyses. Ideally, all patients with DCM should be confirmed to be free of coronary artery disease. In practice, however, coronary arteriography is not routinely performed in all patients with congestive heart failure. Because retrospective studies cannot control the conditions under which patients are recruited or investigated, aside the patients who were once undertaken coronary artery angiography, coronary CT scan or cardiac radionuclide imaging in the other hospitals, there were only 334 patients undertaken coronary artery angiography and 80 patients with positive results in the present study. In addition, to exclude the confusion with ventricular hypertrophy, we defined patients with VCB as QRS duration more than 120 ms. Finally, the patients who creceived ICDs (implantable cardiac defibrillators) or CRTs were not included, and the use of spironolactone and digoxin was higher in the present study.

## Conclusions

The present study indicated that RBBB and IVCD at admission were independent predictors of all-cause mortality in patients with DCM.
